# Identification of hepatitis C virus in the common bed bug – a potential, but uncommon route for HCV infection?

**DOI:** 10.1080/22221751.2020.1780950

**Published:** 2020-06-26

**Authors:** Jiaxin Ling, Thomas Persson Vinnersten, Jenny C. Hesson, Jon Bohlin, Espen Roligheten, Edward C. Holmes, John H.-O. Pettersson

**Affiliations:** a Department of Medical Biochemistry and Microbiology, Zoonosis Science Center, Uppsala University, Uppsala, Sweden; b Anticimex, Stockholm, Sweden; c Infectious Disease Control and Environmental Health, Norwegian Institute of Public Health, Oslo, Norway; d Boligbygg, Oslo, Norway; e School of Life and Environmental Sciences and School of Medical Sciences, Marie Bashir Institute for Infectious Diseases and Biosecurity, The University of Sydney, Sydney, Australia

**Keywords:** Common bed bug, hepatitis C virus, RNA-sequencing, meta-transcriptomics, epidemiology

## Abstract

During an ongoing virome metagenomics project we identified hepatitis C virus (HCV) in a pool of recently blood-fed common bed bug (*Cimex lectularius*) nymphs sampled from domestic residences in Europe. Additional PCR and genomic analysis revealed that the virus was a member of HCV genotype 3A, one of the most prevalent genotypes in Europe. Although the role of the common bed bug in the transmission of human hepatitis viruses remains unclear, our study suggests that it merits additional investigation.

There has been a global resurgence in the common bed bug (Cimicidae, *Cimex lectularius*) [1]. During the early 1900s most large cities in Europe were infested by the parasite [2]. Intense and effective eradication methods, including the use of methyl bromide and DDT, were used widely in the first half of the twentieth century and almost led to the extinction of bed bugs. However, since the relaxation of active control methods bed bug infestations are now widespread in Sweden and many other countries.

The common bed bug is a hematophagous ectoparasite on humans, rarely feeding on other warm bloodied animals. The species has five developmental life stages: four nymphal instars and the imago (adult) stage. During each instar stage the animals require at least one blood meal to develop to the next stage. Following a blood meal, bed bugs retreat to cracks and crevices for 3–5 days for digestion, and eventually molt into the next instar. Bed bugs are also nocturnal and aggregate in concealed harbourages during the day. They become active during the dark hours and are attracted to carbon dioxide, human odours and body heat. At an infested site, the majority of the bed bugs are found close to the food source, particularly areas where humans sleep. However, bed bugs also move around more in heavy infested areas, such that they can be found relatively far from human sleeping areas [3].

As bed bugs numbers have increased dramatically in several parts of the world, we used a meta-transcriptomic (i.e. total RNA-sequencing) approach to determine their virome diversity. Accordingly, bed bugs of all developmental stages were collected from heavy infested inhabited domestic residences in Uppsala and Stockholm, Sweden, and Oslo during 2017, and were found on couches, recliners, bed-sheets, mattresses and box springs. All bed bugs, including nymphs of all stages and imagoes, were collected and kept alive until stored in −80°C. Subsequently, they were separated according to life stage (imagoes or nymphs) and if they had recently fed (i.e. contained visible blood) or not.

In total, 12 pools were created, each pool containing 10 individual bugs (12 × 10): three pools each of unfed imagoes, blood-fed imagoes, unfed nymphs and blood-fed nymphs. All pools were subjected to homogenization, total RNA extraction, RNA-sequencing library preparation and virome analysis as previously described [4]. Briefly, each pool was homogenized followed by total RNA extraction, ribosomal RNA depletion via Ribo-Zero Gold (human-mouse-rat) kit (Illumina) followed by TruSeq total RNA library preparation (Illumina), and finally paired-end (150 bp read-length) sequencing on a single Illumina HiSeq X10 lane. After quality trimming using Trimmomatic v.0.36 (https://github.com/timflutre/trimmomatic), the data was assembled *de novo* using Trinity v.2.5.4 (https://github.com/trinityrnaseq/trinityrnaseq) with default options.

To identify potential RNA viruses, *de novo* assembled contigs were screened using blastn v.2.6.0+ and Diamond v.0.9.15.116 (https://github.com/bbuchfink/diamond), respectively, against the complete NCBI non-redundant nucleotide and protein databases with 1 × 10^−5^ as a cut-off e-value. Following preliminary inspection of the data, one library, BB19, comprising recently blood-fed nymphs and a total sequence output of 65,528,722 reads, was found to have reads that showed strong sequence similarity to hepatitis C virus (HCV) (family *Flaviviridae*). Specifically, we identified eight reads with high sequence similarity (89.7–94.7% nucleotide similarity) to a HCV reference sequence (NC_009824.1) with an estimated abundance (i.e. number of HCV reads per million reads in the library) of 0.12. The presence of these HCV-like reads was further evaluated via an NCBI blastn search to identify the most similar complete genome. Following the similarity search, a reference based mapping approach, using Bowtie2 v.2.3.4 (https://github.com/BenLangmead/bowtie2), was conducted with a HCV genome (KY620846.1) in which reads were mapped to four different genomic regions.

The presence/absence of HCV RNA in the original BB19 total RNA extract and all other 11 RNA pools, respectively, was confirmed using five different sets of PCR-primers, designed using both the identified reads and the KY620846.1 reference as a templates (Supplementary table 1). First, RNA was reversely transcribed to cDNA by using SuperScriptTM III reverse Transcriptase (Invitrogen, USA). Then, separate PCR reactions were performed using Phusion Flash High-Fidelity PCR Master Mix (Thermo Scientific, Lithuania), followed by PCR product clean-up using the QIAquick PCR Purification kit (Qiagen, Germany) and Sanger sequencing.

By using the reads and the PCR products, a partial HCV majority consensus genome was computed, which was then used to retrieve 11 representative closely related sequences with high sequence similarity and eight different genotype outgroup sequences retrieved from GenBank. After sequence alignment, using Mafft v.7.271 (https://github.com/nesi/applications/wiki/MAFFT), a maximum likelihood phylogenetic tree was estimated using IQ-TREE v.1.6.12 (https://github.com/Cibiv/IQ-TREE), employing the best-fit TIM2+F + R4 model of nucleotide evolution and annotated in FigTree v.1.44 (http://tree.bio.ed.ac.uk/software/figtree/).

The phylogenetic analysis revealed that the HCV sequences originally derived from the bed bug RNA sequence library (BB19) fell within a clade of HCV genotype 3A sequences (100% bootstrap support), and were most similar to a sequence (KY620688) sampled from the blood of a human with unknown location information. The phylogenetic tree and overview of HCV reads and PCR products in relation to the reference genome is provided in Figure 1.

**Figure 1.**
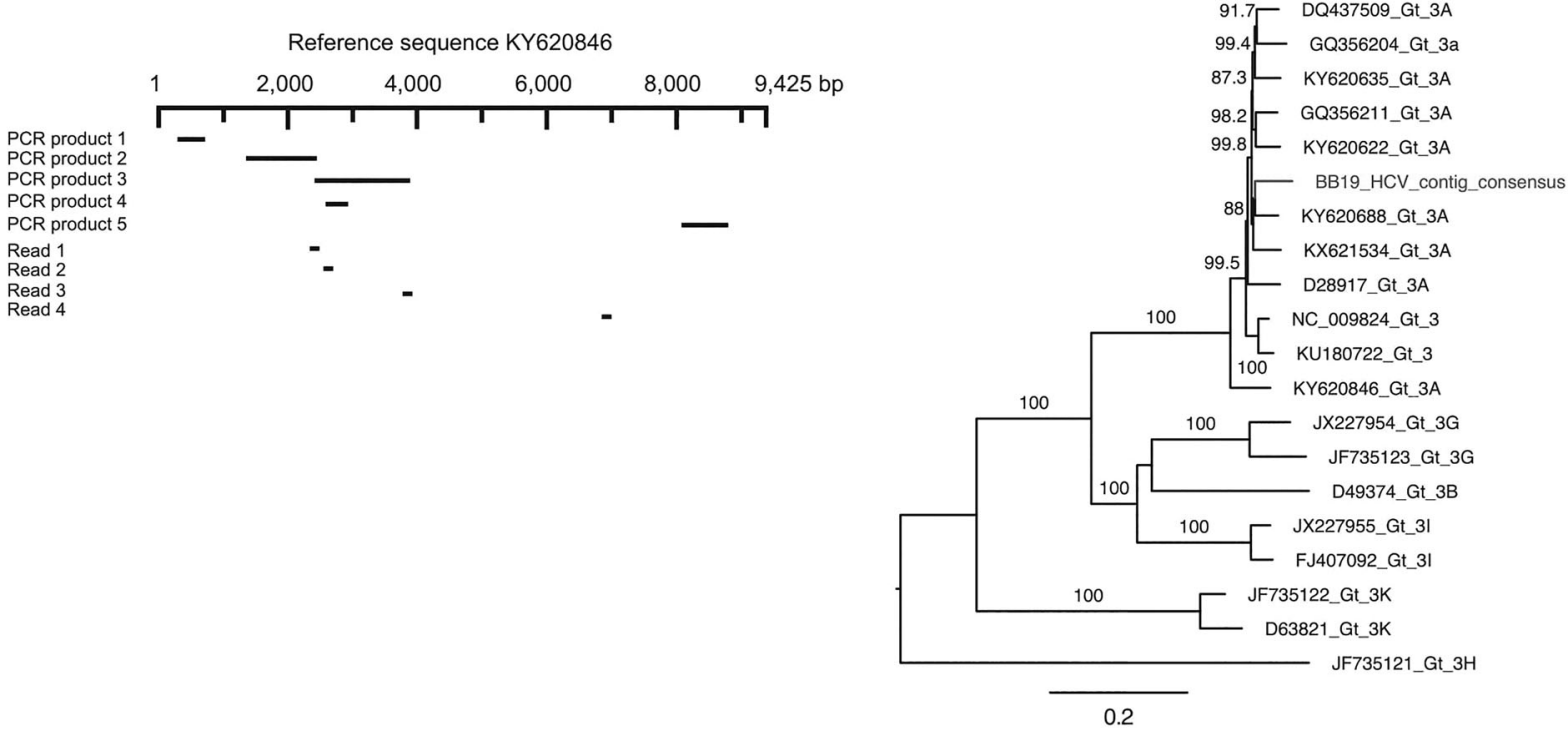
Mapping of HCV reads and PCR products and phylogenetic analysis of HCV. Reads and PCR products were mapped and aligned against the most similar reference genome (KY620846), which were subsequently used to compile a partial consensus genome. This partial genome sequence was then aligned with a background set of HCV sequences and a maximum likelihood phylogenetic analysis was performed. Node robustness was assessed using Shimodaira–Hasegawa-like branch support values. The phylogenetic tree is mid-point rooted for clarity only. The HCV contig is available via NCBI GenBank (accession number: MT254535) and the raw sequence data is available via NCBI Short Read Archive (BioProject ID: PRJNA613931).

To the best of our knowledge this is the first time HCV has been detected in the common bed bug collected from the field. The HCV genotype 3 identified is one of the most common world-wide, particularly in high-income countries, likely due to its association with people who inject drugs and migration from high-prevalence countries [5]. In a previous study where bed bugs were experimentally infected with HCV, the virus could not be detected after feeding from an infectious source [6]. In contrast, we show here that it is possible to detect HCV in newly blood-fed bed bugs at least within the first 24 h after feeding an infectious meal. That is, the HCV RNA was likely only present in the blood meal and not taken up by the bedbug itself. Furthermore, the abundance of HCV in the library was relatively low (0.12 HCV reads per million reads), such that the virus may not have actively replicated in the bed bug and/or that only a single bedbug had taken an HCV positive blood meal.

A key outstanding question is whether bed bugs can transmit this virus to humans? There is a possibility, albeit relatively low, that the virus can be transferred via mechanical transmission, for example following an infectious bite [7]. However, while multiple bacterial, viral and parasitic pathogens have been identified in bed bugs (reviewed in [8–10]), and humans do develop an immune response and dermatological reactions [8,11], there is currently no evidence for any causal relationship between bed bug bites and the transmission of human pathogens [12]. It is therefore possible that bed bugs lack the vectorial competence to transmit human pathogens. Indeed, given the number of bed bug infestations globally it is likely that any role they play in pathogen transmission among humans would have been documented already [13].

Following meta-transcriptomic next-generation sequencing and PCR confirmation we identified HCV in a pool of 10 recently blood-fed bed bug nymphs. If bed bugs were to be involved in virus transmission, the greatest risk of infection in many European countries would involve people who inject drugs, have a relatively high prevalence of both HBV and HCV [14,15], and who reside in high-density living areas. Such a social situation provides opportunities for interrupted feeding and subsequent feeding off a new host, increasing the risk of both potential mechanical and biological transmission. Hence, we suggest that our study warrants a further investigation of the role of the common bed bug as a potential vector for the transmission of HCV.

## Supplementary Material

Supplementary_table_1.docx

## References

[1] Doggett SL, Dwyer DE, Peñas PF, et al. Bed bugs: clinical relevance and control options. Clin Microbiol Rev. 2012;25:164–192. doi: 10.1128/CMR.05015-11 22232375 PMC3255965

[2] Naylor R, Balvín O, Delaunay P, et al. The bed bug resurgence in Europe and Russia. In: Doggett SL, Miller DM, Lee C-Y, editor. Advances in the biology and management of modern bed bugs. Chichester: Wiley; 2018. p. 59–68.

[3] Wang C, Saltzmann K, Chin E, et al. Characteristics of Cimex lectularius (Hemiptera: Cimicidae), infestation and dispersal in a high-rise apartment building. J Econ Entomol. 2010;103:172–177. doi: 10.1603/EC09230 20214383

[4] Pettersson JH-O, Shi M, Bohlin J, et al. Characterizing the virome of Ixodes ricinus ticks from Northern Europe. Sci Rep. 2017;7:1–7. doi: 10.1038/s41598-016-0028-x 28883464 PMC5589870

[5] Messina JP, Humphreys I, Flaxman A, et al. Global distribution and prevalence of hepatitis C virus genotypes. Hepatology. 2015;61:77–87. doi: 10.1002/hep.27259 25069599 PMC4303918

[6] Silverman AL, Qu LH, Blow J, et al. Assessment of hepatitis B virus DNA and hepatitis C virus RNA in the common bedbug (Cimex lectularius L.) and kissing bug (Rodnius prolixus). Am J Gastroenterol. 2001;96:2194–2198. doi: 10.1111/j.1572-0241.2001.03955.x 11467652

[7] Jupp PG, McElligott SE, Lecatsas G. The mechanical transmission of hepatitis B virus by the common bedbug (Cimex lectularius L.) in South Africa. S Afr Med J. 1983;63:77–81. 6849170

[8] Goddard J, deShazo R. Bed bugs (Cimex lectularius) and clinical consequences of their bites. JAMA. 2009;301:1358–1366. doi: 10.1001/jama.2009.405 19336711

[9] Delaunay P, Blanc V, Del Giudice P, et al. Bedbugs and infectious diseases. Clin Infect Dis. 2011;52:200–210. doi: 10.1093/cid/ciq102 21288844 PMC3060893

[10] Adelman ZN, Miller DM, Myles KM. Bed bugs and infectious disease: a case for the arboviruses. PLoS Pathog. 2013;9:e1003462. doi: 10.1371/journal.ppat.1003462 23966852 PMC3744395

[11] Delaunay P, Blanc V, Dandine M, et al. Bedbugs and healthcare-associated dermatitis, France. Emerging Infect Dis. 2009;15:989–990. doi: 10.3201/eid1506.081480 PMC2727309 19523318

[12] Lai O, Ho D, Glick S, et al. Bed bugs and possible transmission of human pathogens: a systematic review. Arch Dermatol Res. 2016;308:531–538. doi: 10.1007/s00403-016-1661-8 27295087 PMC5007277

[13] Doggett SL. Bed bugs and infectious diseases. In: Doggett SL, Miller DM, Lee C-Y, editor. Advances in the biology and management of modern bed bugs. Chichester: Wiley; 2018. p. 117–125.

[14] Pettersson JH-O, Myking S, Elshaug H, et al. Molecular epidemiology of hepatitis B virus infection in Norway. BMC Infect Dis. 2019;19:1–7. doi: 10.1186/s12879-019-3868-8 30845915 PMC6407267

[15] Wiessing L, Guarita B, Giraudon I, et al. European monitoring of notifications of hepatitis C virus infection in the general population and among injecting drug users (IDUs) – the need to improve quality and comparability. Euro Surveill. 2008;1–5. 10.2807/ese.13.21.18884-en 18761969

