# Supplementary material for: Identification of hepatitis C virus in the common bed bug – a potential, but uncommon route for HCV infection?
Source: Emerg Microbes Infect. 2020 Jun 26;9(1):1429–31. doi: 10.1080/22221751.2020.1780950 (PMC7473286; doi:10.1080/22221751.2020.1780950)
Supplement: Supplementary_table_1.docx [file TEMI_A_1780950_SM4612.docx]

**Supplementary table 1. Primers used in the study**

| Primer* | Sequence 5’–3’ | Product length (bp) |
| --- | --- | --- |
| HCV3F_269_287^b^ | CCTTGTGGTACTGCCTGAT | 476 |
| HCV3R_728_744^b^ | GGGATGTACCCCATGAG |  |
| HCV3F_1285_1304^b^ | GGCTTGGGATATGATGATGA | 1125 |
| HCV3R_2391_2409^a^ | AATGCAGGCATAGGCGTGA |  |
| HCV3F_2300_2319^a^ | GGGGAGCGCTGCGAYATYGA | 275 |
| HCV3R_2553_2574^a^ | ATCAGCATCAGCCAGATGGCAA |  |
| HCV3F_2300_2319^a^ | GGGGAGCGCTGCGAYATYGA | 1598 |
| HCV3R_3876_3897^a^ | CTAAAGATTCCCGCAACATGCC |  |
| HCV3F_8042_8061^b^ | ATGGCGAAGAACGAGGTGTT | 749 |
| HCV3R_8771_8790^b^ | GCATCGCGGGTGAGGTAATA |  |

*Nucleotide positions correspond to the reference sequence HCV3 isolate P117210 (Genbank Accession no. KY620846).

(a) primers are designed according to HCV reads.

(b) primers are designed correspond to HCV conserved regions.
